# Work from Home Challenges of the Pandemic Era in Hong Kong: A Stimulus-Organism-Response Perspective

**DOI:** 10.3390/ijerph19063420

**Published:** 2022-03-14

**Authors:** Tai Ming Wut, Stephanie Wing Lee, Jing (Bill) Xu

**Affiliations:** College of Professional and Continuing Education, The Hong Kong Polytechnic University, Hung Hom, Hong Kong; s.lee@cpce-polyu.edu.hk (S.W.L.); bill.xu@cpce-polyu.edu.hk (J.X.)

**Keywords:** work from home, COVID-19, stimulus-organism-response model, physical isolation, psychological isolation, sense of belonging, teamwork climate, work engagement

## Abstract

Hong Kong is an international city where almost all the companies did not have a WFH policy before the pandemic since it is a very small place. During the pandemic period, Hong Kong governments, major banks and large private organizations have adopted WFH policy. The purpose of this article is to examine impact of work from home (WFH) practice on work engagement with the company during the pandemic period in Hong Kong. According to a stimulus-organism-response model, this study explores the dark side the WFH arrangement during the pandemic period. Convenience sampling method was used to collect 206 valid responses from individuals who were working from home in Hong Kong. Partial least squares structural equation modelling (PLS-SEM) was used in the analysis of data. It was found that teamwork climate is negatively associated with physical isolation and sense of belonging is negatively associated with psychological isolation. Work engagement was impaired. Affective social presence may not be so easily established through virtual ways. Virtual informal gatherings, such as virtual breakfasts, lunch or tea breaks where work-related matters are not discussed, could be arranged.

## 1. Introduction

The sudden outbreak of COVID-19 in early 2020 forced many companies to introduce work from home (WFH) measures. Global tech giants are early adaptors of WFH arrangements, and with the persistence of the pandemic, many large corporations have extended WFH arrangements for their office staff. Apple, Reuters and Amazon announced that their staff would continue working from home until early 2021, whereas Google and Facebook announced a WFH extension up to mid-2021. Several multinational corporations in other industries have adopted similar policies, including the leading music entertainment groups Universal, Warner and Sony, the digital music service provider Spotify, and the Royal Bank of Scotland. Indeed, all of which have extended their WFH policies to 2021. As the pandemic may persist for an indefinite period, some companies do not impose a fixed duration for WFH. For example, Spotify would open its offices on a city-by-city basis in accordance with the guidelines of local governments, whereas Mastercard currently has no plans of formally returning to office [1].

Approximately 40% of jobs can be carried out from home, but this share varies across countries and sectors. ‘Financial and insurance activities’, ’information and communication’ and ’education’ are the top three sectors with highest potential for WFH, whereas ‘agriculture, forestry and fishing’, ‘accommodation and food services’ and ‘construction’ are the least feasible to engage in WFH [2]. Such variation also explains why WFH arrangements are concentrated in developed countries and amongst relatively large firms given that industries in developing countries, which mostly comprise small production businesses, have poor capability to carry out WFH [3]. A Norwegian report shows that when the government promotes WFH in an attempt to mitigate the spread of COVID-19, the disadvantaged groups in the labour market, including immigrants, young workers and single parents, will greatly suffer given that they are less likely to have jobs that can be performed remotely [4].

Many studies on WFH that were published after the COVID-19 outbreak have mainly focused on the economic and social impacts of related WFH polices. A German study found that WFH can reduce output losses induced by infection and fatalities related to COVID-19 and suggested that WFH arrangements should be maintained as long as possible [2]. The wave of WFH has also changed the population formation of cities. For instance, some US residents formerly working in central business districts have moved to periphery areas, thereby easing traffic congestion and reducing the average real estate prices in metropolitan areas [5]. Additionally, less time pressure and fewer work-family conflicts, thus a better quality of life was reported [6,7].

However, workers in WFH settings are socially isolated and do not have opportunities to engage in social interactions unlike in office settings. Therefore, loneliness has emerged as a key topic in WFH studies that warrants exploration. As they continue working from home, employees could have a reduced sense of belonging (SOB) to their organisations and may even think of quitting [8]. To compensate for reduced physical interactions in WFH settings, companies have to search for alternative ways to institutionalise trust, which may require commitment to relationship building and ongoing communication [9]. For instance, managers may allot some time for informal communication at the beginning of their team meetings or organise virtual office parties by sending food or ‘care packages’ to their team members for their enjoyment whilst attending videoconferences. Leaders should also acknowledge the stress that their employees may face in a WFH setup and offer them encouragement and emotional support accordingly [8].

Whilst people who always work away from physical offices can keep in touch with their immediate supervisors and other colleagues, they may feel isolated. These people lack an official reporting line to their colleagues and are used to engage in informal communications with their colleagues in a physical office. The effectiveness of teamwork may also be compromised in this setting. As a result, work engagement can be adversely affected, and organisational sustainability, which is built on the values of altruism and empathy, can be jeopardized [10].

This study aims to investigate how WFH policy affects work engagement in a corporate setting in Hong Kong. Does WFH policy affect employees’ work engagement? Hong Kong is an international city, which does not have WFH policy before the pandemic unlike United States or some European countries. It is a very small city with around one thousand square meters thus we might travel around within one to two hours. We utilised the stimulus-organism-response (SOR) theory [11] to propose our basic research model. The rest of the paper is structured in five sections. Section 2 reviews the pertinent prior studies and theories, builds a conceptual framework and proposes the hypotheses. Section 3 discusses the research methodology which is followed by the results section. Discussion and conclusion sections are presented at the end.

## 2. Literature Review

### 2.1. Stimulus-Organism-Response (SOR) Theory

The stimulus-organism-response (SOR) theory is a well-known method to analyse various variables into three categories: stimulus, organism and response. Stimulus is external or environmental shock that affecting some other constructs. Organism represents intermediate state and response is represented by a behaviour [11]. It would be similar to the logic of input–process–output [12]. It was a popular psychology theory to explain customer behaviour [13]. It has been used in other management and human resources fields [14]. We thereby suggest a basic model based on the S-O-R theory to examine how WFH policy (stimulus) affects employees’ sense of belonging/teamwork climate, which in turn influence work engagement.

Important variables including ‘psychological isolation’, ‘physical isolation’, ‘teamwork climate’, ‘sense of belonging’ and ‘work engagement’ are explained from Section 2.2 to Section 2.6, respectively. Then, paragraphs leading to hypotheses are arranged. Section 2.7 refers to the relationship between psychological isolation and physical isolation. Section 2.8 refers to the relationship between physical isolation and teamwork climate. Relationship between psychological isolation and sense of belonging is found in Section 2.9. Last but not least, relationship between teamwork climate, sense of belonging and work engagement is discussed in Section 2.10.

### 2.2. Psychological Isolation

Psychological isolation is an emotional state where one cannot connect himself/herself to others [15]. An employee may feel disconnected from his/her colleagues when working from home. Compared with those working in face-to-face environments, those employees working from home may be less influenced by their colleagues and have relatively weaker social networks, which are usually established through informal gatherings, such as chats, lunches, tea breaks and happy hours [16]. A prior survey shows that the majority of the home-based workers in the UK (90%) have begun to consider socialisation time more important than ever and that 63% of these respondents have felt isolated since they started working from home [17].

In functional terms, isolation can be divided into ‘professional isolation’ (reduced opportunities for promotion, rewards, or personal development) and ‘social isolation’ (resulting from limited interactions with co-workers), with the former being relevant to the latter [18]. Full-time teleworkers develop individualisation and a high level of social disconnection from office-based workers largely due to the limited chances available for social interactions [19]. Nevertheless, WFH may reduce stress from commuting and avoid the negative aspects of working in an office environment, such as constant supervision, office politics, harassment, sexism and hierarchy [19].

### 2.3. Physical Isolation

Physical isolation refers to a state where one feels separated from his/her colleagues [20]. This type of isolation becomes significant when WFH is implemented continuously over time. Before the popularisation of WFH, some scholars have investigated the psychological aspect of telecommuting. A theoretical paper summarised those organisational factors that increase the likelihood of telecommuting employees to develop subjective feelings of social isolation, which hinder them from identifying team norms and/or values. These factors include the absence of identifiable artefacts of status (e.g., office space, formal attire, and lack of time structure), lack of opportunities for experience sharing and work fragmentation [21]. Meanwhile, offsite workers often use mobile apps, such as instant messaging, to interact with their colleagues outside their designated work time and space in order to reduce their feelings of social isolation [22].

Private sector managers frequently highlighted the importance of interpersonal networking in establishing familiarity and camaraderie. However, government supervisors and employees seem to place relatively less importance on face-to-face contact, which may not be considered instrumental in career advancement in public sector [18].

Team virtuality was distinguished from task virtuality based on the degree of remote work. The former can be viewed as semi-remote work where employees lack physical communication with their colleagues (e.g., despite not physically interacting with their team members, some salespersons still have to meet their clients face to face), whereas the latter concerns the overall exposure of employees and their colleagues in the workplace. When these employees physically interact with those colleagues who they deem to have important roles in fulfilling their tasks, they feel SOB, social support and friendship [23].

### 2.4. Teamwork Climate

Teamwork climate is defined as agreement of interpersonal relationship norms [20]. Communication, monitoring and backup are key parts of teamwork. Communication includes an active exchange amongst team members; monitoring refers to evaluating the performance or providing feedback to colleagues; and backup refers to the behaviour of helping other team members in performing their tasks [24]. Leadership is crucial in guiding team members towards achieving collective goals, whereas teamwork requires team orientation, which encompasses positive attitudes and self-awareness of individual members, and contributes to group cohesiveness [24].

Workplace friendships includes commitment from both sides, trust and shared values or interests amongst colleagues; this type of relationship not only increases the availability of support and resources that help workers finish their tasks but also facilitates organisational changes and enhances productivity [25]. Unlike most types of friendships, workplace friendships often involve relations amongst people with vast differences in their age, status, or gender (e.g., between supervisors and subordinates and between young and elderly workers). However, given that workplace relations are solely based on work-related interests, such as projects or shared locations, they tend to disappear along with the aforementioned factors unless a new common ground emerges [25].

A survey of R & D teams in the top 100 scientific firms in Taiwan shows that workplace friendships can promote innovative behaviour and discourage job burnout [26]. Team leaders should allow teammates to have social activities. Moreover, interdepartmental events that encourage casual interactions may facilitate information sharing [26].

### 2.5. Sense of Belonging

Sense of belonging (SOB) can be defined as “the experience of personal involvement in a system or environment so that persons feel themselves to be an integral part of that system or environment” [27]. Belonging is described as a basic human need and argued that emotional breakdowns are closely connected to an unfulfilled need for belonging [28]. Sense of belonging is basic to personal well-being, and individuals with high SOB are embedded in a stable social network [29]. A previous study on college students shows that SOB is closely related to both social and psychological functioning [30]. A comprehensive understanding of SOB covers different contexts to which individuals are exposed, such as family, work, school and church [31].

SOB may differ across cultures. For instance, collectivistic cultures place more emphasis on belonging to a social group compared with independent cultures [32]. In hierarchical societies with a high-power distance, citizens are taught to obey authority given their high status, whereas low-power-distance egalitarian societies encourage equality and are thereby more likely to cultivate friendly relationships [29]. Gender may also influence SOB. A survey of school children reveals that girls have significantly higher peer acceptance ratings, and thereby higher SOB, compared with boys [31].

SOB has a significant positive relation with overall job satisfaction, praise, control, co-workers, work schedule and engagement [33]. The physical environment of a workplace can also affect SOB [34]. Moreover, human resource personnel can use social media as a new communication medium to interact with their employees and to improve their organisational commitment and SOB to their company [35].

### 2.6. Work Engagement

Work engagement is defined as a “positive, fulfilling work-related state of mind that is characterized by vigor, dedication, and absorption” [36]. This factor is important for those companies that aim to retain employees with high work engagement. A higher work engagement corresponds to more opportunities for companies to grow and accumulate more profit [37].

Working in a virtual setup provides employees with a high level of autonomy and implies a greater reliance on their self-discipline. The ability to work without stop is also based on the physical conditions at home and the young family [17]. A European online survey on job satisfaction reveals that 48.8% of the respondents have been working from home full-time. In terms of perceived performance, 71.2% of these respondents feel less productive in a WFH setup than in the office, whereas 58% have developed some feelings of guilt [38]. Office workers might worry about if he or she cannot finish the task on time. They might hesitate to chase their co-workers in WFH mode [39]. Relevant considerations should be used to reduce these psychological issues and to prevent the long-term psychological consequences of WFH, such as anxiety and depression.

The association between the work engagement and performance of employees has been comprehensively examined over the past 20 years [40]. For theoretical support, drivers of work engagement include ‘job resources’ (e.g., social support from colleagues and supervisors, performance feedback, skill variety, autonomy and learning opportunities) and ‘personal resources’ (e.g., perceived ability of individuals to control their environment) [41,42]. With these resources, engaged employees can demonstrate performance improvements because they tend to “experience positive emotions and better health, can create their own job and personal resources and can transfer their engagement to others” [41].

### 2.7. Relationship between Psychological Isolation and Physical Isolation

Telecommuting brings about psychological isolation and physical isolation amongst colleagues, clients and other people affected by the pandemic. Psychological isolation is a condition of emotional unfulfillment that results from the lack of meaningful connections, support and interactions and has been linked to physical separation given that employees are working in locations other than the office, including but not limited to client sites, homes, cafes and hotels [43].

Leadership style may affect the psychological influence of physical separation at work. For example, some telecommuters argue that online relationships can reduce the influence of power hierarchy. However, loneliness and frustration resulting from the lack of face-to-face interactions may have critical roles in the career development of individuals and subsequently have negative effects on the quality of peer relationships [44].

**Figure 1:**
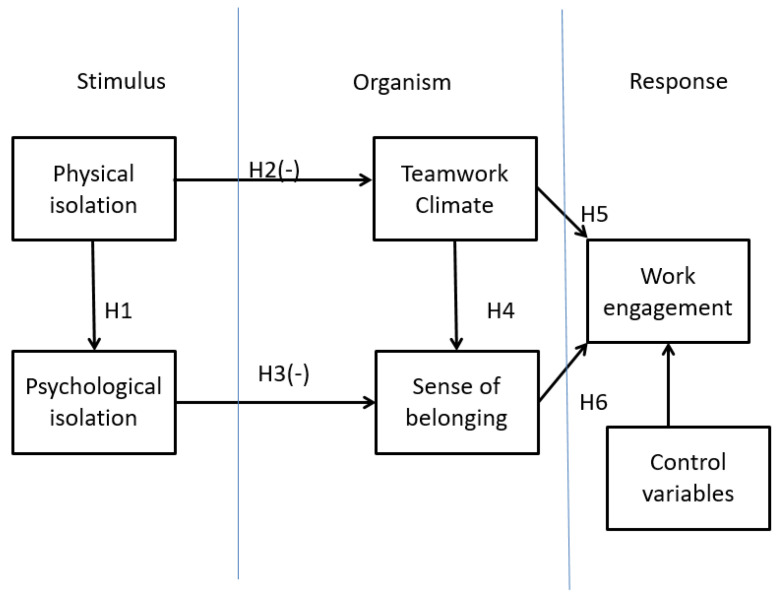
Conceptual model. (Source: authors).

The full research model of this study is presented in Figure 1 along with the proposed hypotheses. As employees continue working from home during the pandemic, they may perceive intensified feelings of psychological isolation.

**Hypothesis** **1.** *The physical isolation of employees is positively associated with their perceived psychological isolation*.

### 2.8. Relationship between Physical Isolation and Teamwork Climate

Electronic communication technologies enable the formation of a ‘virtual teamwork’ across different geographical locations in a fully mediated online context. Through these technologies, team members living in different time zones can participate in video conferences, share files on cloud platforms and document their work progress [45]. Employees have more opportunities to interact with one another when they are working under the same roof. By contrast, even though computer-mediated tools enable employees to communicate with one another irrespective of their locations, they cannot fully replace the low-virtuality spontaneous and informal interactions in face-to-face encounters, which help establish and maintain meaningful and lasting relationships amongst co-workers [43].

Virtuality is formed by a complex construct that covers certain dimensions, including the degree of electronic mediation, synchronicity of communication and geographic dispersion; the level of virtuality influences the effectiveness and related psychological processes of teams, such as leadership, trust, well-being, social exchange and social exclusion [45,46].

Prior to the onset of COVID-19, virtual teamwork was mainly considered an alternative working option that provides employees with high flexibility; however, with the spread of COVID-19, virtual teamwork has become a necessity for organisations to continue their operations [45]. Communication serves four major functions within an organisation, namely, “control, motivation, emotional expression and information”, and virtual communication may not be an excellent substitute that can fulfil these functions [44]. To promote social connectedness in virtual teamwork contexts, regular informal meetings, such as virtual coffee breaks, can be organised for mutual support; synchronous communication with visual information can also transmit non-verbal cues that help maintain trust amongst team members [45].

As employees continue working from home, their teamwork climate may be affected as they are not working in the same office [47].

**Hypothesis** **2.** *The physical isolation of employees is negatively associated with their perceived teamwork climate*.

### 2.9. Relationship between Psychological Isolation and Sense of Belonging

Human beings are “social by nature and have the innate desire to develop positive emotional connections with others” [48], achieve a sense of acceptance through frequent interactions and be recognised as part of their teams. Physical isolation negatively affects organisational identification, especially for new employees at the initial stage of establishing a psychological relationship with their companies [20]. Commitment refers to the attachment that an individual feels to a collective entity, in this case, a company [49].

Although telecommuters show continuance commitment to their employers because of the perceived benefits (e.g., travel time and energy savings or reduced job security), their affective commitment is adversely influenced by their psychological isolation [43]. Feelings of isolation and impaired SOB are the two major disadvantages of telecommuting; at the societal level, the increasing number of telecommuters may create a detached, autistic society where individuals are isolated from one another [50].

Therefore, a high degree of psychological isolation is expected to reduce the SOB of employees to their organisations.

**Hypothesis** **3.** *The perceived psychological isolation of employees is negatively associated with their perceived SOB*.

### 2.10. Relationship between Teamwork Climate, Sense of Belonging and Work Engagement

Relational cohesion theory proposes that “the positive affective connections amongst individuals may contribute to their emotional and normative commitment (sense of embeddedness and responsibility)” to the organisation [49]. Under the context of COVID-19, the concept of team-perceived virtuality was proposed, which comprises two dimensions, namely, collectively experienced distance, which refers to the awareness from both sides of emotional in-accessibility and may result in cold, unfriendly, unaffectionate and estranged relationships amongst teammates, and cannot receive information fully, which are characterised by the collective perceptions of poor information exchange held by team members [47].

Managers may face challenges in transmitting corporate culture to telecommuting employees given that these employees are not exposed to the beliefs and values of their organisations in physical settings [51]. Relationship-oriented companies do not have formal rules and rely on monitoring by supervisors. The supervision will be weakened by work from home measures. It has been suggested that procedures such as those adopted in task-oriented organisations would be needed [51].

Based on the above arguments, a greater level of teamwork climate corresponds to a greater SOB to the organisation [43].

**Hypothesis** **4.** *The perceived teamwork climate of employees is positively associated with perceived SOB*.

Hypotheses 5 and 6 address the path relationships from teamwork and SOB to work engagement, respectively.

**Hypothesis** **5.** *The perceived teamwork climate of employees is positively associated with perceived work engagement*.

**Hypothesis** **6.** *The perceived SOB of employees is positively associated with perceived work engagement*.

## 3. Methodology

Quantitative method was used in this study. A questionnaire survey was conducted to ask standardised questions and to facilitate the data collection with the assistance of a web-based tool. English and Chinese are the main languages used in the survey. Back translation was performed to ensure that the questions share the same meanings across the two languages.

### 3.1. Measurement

All variables in the conceptual framework were measured by using existing constructs on a seven-point Likert scale (1 = strongly disagree; 4 = neutral; and 7 = strongly agree) (See Appendix A). The work engagement scale was adapted from the nine-item Utrecht work engagement scale [52]. This scale consists of three sub-scales, namely, vigour (e.g., ‘I feel strong and vigorous in my job’), dedication (e.g., ‘I am enthusiastic about my job’) and absorption (e.g., ‘I am immersed in my work’)” [52]. This scale has been tested extensively and can be used in organisational behaviour studies.

The psychological isolation scale, originally called the professional isolation scale, was adapted from the seven-item scale of Golden et al. [15]. Physical isolation was objectively measured by a single item (‘How many days per week are you engaged in WFH this year or in the previous month’) [20]. Teamwork climate was measured by using the six-item scale of Sexton et al. [53], which was originally administered in a hospital setting. Minor changes in the wordings of the items in the adaptation of the established scales. For example, in the scale of teamwork climate, workplace was used instead of clinical area. Face validity was thus preserved.

Age, gender, job level, tenure with the company, industry type and company size were added in our research model as control variables to rule out other possible explanations for the proposed relationships. If an employee is new to company, then s/he may require more informal support from his/her colleagues. If an employee has been working for his/her company for many years, then s/he may be familiar with most colleagues and is unlikely to feel isolated. A larger company has greater flexibility to arrange events for employees. By contrast, employees working for small companies usually take on multiple roles and are unable to fully implement WFH practices easily.

Partial least squares structural equation modelling (PLS-SEM) was used in the analysis of data instead of covariance-based structural equation modelling due to small sample size and non-normal data [54]. With an 80% statistical power, the sample size is a hundred at 5% significance level with the two arrows pointing at a construct maximum in this study [54]. SmartPLS 3.0 software was used to test the model.

### 3.2. Data Collection and Respondents Background

The data using a survey format were collected from employees of various industries, whose responses were gathered in August 2020 by using Google Forms. Convenience sampling method was used. Potential respondents were approached via email with detailed information of the study and consent form. Written consent was obtained from all respondents. The survey was anonymous in nature. Participation was entirely voluntary. Respondents were told that they could withdraw at any time without negative consequences. No incentive was provided for participants. All participants were over the age of 18. A total of 250 electronic mails were sent out. Overall, 210 responses were received, of which 4 had essential information missing and were therefore discarded. The response rate was 84%.

The sample size was 206. In total, 53.4% were men and 46.6% were women in the sample. In terms of age, 31.1% of these respondents were aged between 41 and 50 years, 38.8% were aged between 18 and 30 years and 19.4% were aged between 31 and 40 years. A total of 40.8% of the respondents were professionals, 16.5% were engaged in retailing and customer service, 12.6% were engaged in trading and logistics and 15.5% were engaged in financial services. In terms of company size, almost half of the respondents (39.8%) were working in large companies having more than 100 employees, and 38.8% were working in medium-sized companies with 21 to 100 employees. In terms of tenure, around 23% of the respondents had been working for more than 10 years in their companies, 13% had been working for 5 to 10 years, 22% had been working for 2 to 5 years and 32% had been working for half a year to less than 2 years. In total, 38.8% of the respondents were at entry level position, 27.2% were at supervisory management level and 25.2% were at middle management level (Table 1). In sum, almost all respondents possessed experience in working for large- or medium-sized organisations.

Around a quarter of the respondents were working in an office, another quarter were working from home entirely and all the other respondents were working from home for one to four days every week depending on the nature of their job. For instance, those employees working in a retail job were working in the frontline or in the workplace, whereas those employees engaged in professional services, such as translation, can work from home on a full-time basis.

## 4. Results

### 4.1. Assessment of the Reflective Measurement Models

In total, 206 valid responses were obtained for the study. Information on exogenous and endogenous variables were received from the same participant. Common method bias needed to be addressed. Herman one factor test was employed to test common method bias [55]. The total variance explained was well below the 50% threshold. Thus, common method bias is not a problem in the study.

Table 2 shows the four constructs model’s reflective measurement model assessment. Firstly, the indicator loadings were compared with the recommended guidelines of 0.708. Some items were dropped for having loadings below 0.708. Two of these items were from teamwork and SOB, one item was from work engagement. Meanwhile, one item from psychological isolation and another item from work engagement were retained for further analysis despite having loadings that were marginally below the threshold. More than 50% of the variances in the indicators were explained and obtained satisfactory item reliabilities. None of the item loadings exceeded 0.90 to avoid redundancy amongst the indicators [56].

Secondly, the Cronbach’s reliability (0.797 to 0.905) and composite reliability (0.866 to 0.924) of all constructs exceeded the recommended standard, thereby forming a satisfactory to good result. Thirdly, all the constructs’ “Average Variance Extracted” (AVE) measures ranged from 0.605 to 0.645, which exceeded the suggested cut-off point of 0.50 and suggested that the constructs explained around 80% of the variance of related items and demonstrated sufficient convergent validity. Lastly, the HTMT scores were below 0.85 (Table 3), thereby suggesting that all constructs were reliable and valid. The structural model was then assessed.

### 4.2. Assessment of Structural Model Fit

The structural model was validated with good results. Firstly, the R2 values of psychological isolation, SOB, teamwork and work engagement were 0.058, 0.453, 0.012 and 0.497, respectively. Therefore, 5.8% to 49.7% of the variances were explained, indicating a weak to moderate result. Secondly, the Q2 values ranged from 0.004 to 0.272, indicating the small to medium predictive relevance of the path model. Finally, all f2 effect sizes of the predictor construct ranged from 0.012 to 0.445, indicating that the dependent variable had small to large effect sizes.

**Figure 2:**
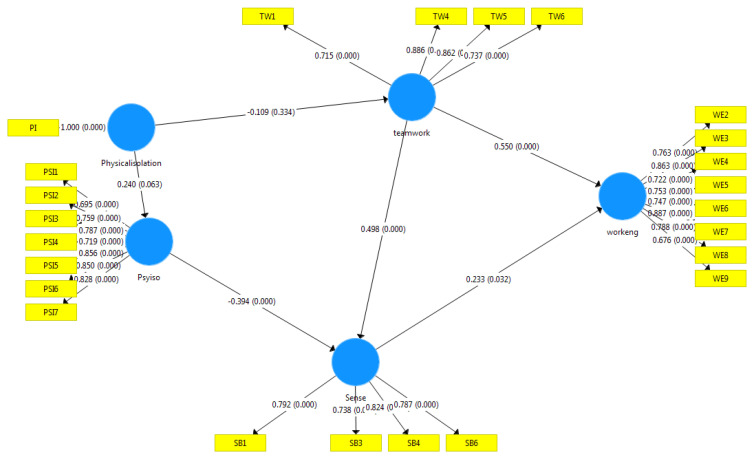
PLS Structural Model (Source: authors).

The path coefficients and t-value were evaluated by conducting bootstrap analysis with 5000 subsamples for the 206 cases. Figure 2 shows the PLS model results. All relationship paths were significant, except the one from physical isolation to teamwork. Therefore, our proposed structural model was well supported.

### 4.3. Results of Hypotheses Testing

Work engagement was the outcome of the conceptual model, whereas teamwork climate and SOB were considered the intermediaries. The following hypotheses testing results highlighted the direct relationships amongst these constructs.

Hypothesis 1 proposes a relationship between physical and psychological isolation. As shown in Table 4, this hypothesis was marginally supported (*p* = 0.063). Hypothesis 2 proposes a negative relationship between physical isolation and teamwork climate. The coefficient (−0.109) is negative and insignificant. Hypothesis 3 proposes a negative relationship between psychological isolation and SOB, which was supported as expected. Hypothesis 4 proposes a relationship between teamwork climate and SOB, which was supported as expected as shown in Table 4. Hypotheses 5 and 6 propose that teamwork climate and SOB are related to work engagement, respectively, both of which were supported. Those hypotheses provide a useful mechanism from physical isolation, through teamwork climate and sense of belonging, to work engagement. The study provides empirical evidence for the proposed model. Teamwork climate has a direct effect on work engagement. Additionally, teamwork climate has an indirect effect on work engagement via sense of belonging.

## 5. Discussion

All the hypotheses in the study were confirmed except Hypothesis 2. Hypothesis 1 proposes a positive relationship between physical and psychological isolation. As expected, a significant association was observed between these two types of isolation. In other words, physical isolation was associated with psychological isolation. As people continue working from home, they may begin feeling uncomfortable. This result is consistent with the findings reported in the literature [43]. With the duration of WFH being longer, there is higher psychological isolation. Employees feel greater level of loneliness on remote work. Thus, swapping of remote and office work could be considered.

Hypothesis 2 proposes a negative relationship between physical isolation and teamwork climate and suggests that a higher degree of physical isolation corresponds to a lower degree of teamwork climate. Teamwork climate is formed mostly from informal meetings such as breakfast, tea or night snack. In contrast to the literature, this hypothesis was not supported by the data, which were collected after the onset of the pandemic during which employees have become used to working from home for extended periods of time. By this time, these employees have already understood how they can use technology to keep in contact with their colleagues. Therefore, teamwork climate is not affected by working from home for extended periods. Here, the construct teamwork climate refers to the formal virtual meetings. There are tools that people could cooperate to complete a job easily without face-to-face contact.

Hypothesis 3 proposes a negative relationship between psychological isolation and SOB. Consistent with the literature [19,43], this hypothesis was supported by the data. Employees with higher psychological isolation, have less sense of belonging to the company. SOB refers one desired to have an acceptance from the company [50]. Employers need to take more initiatives to show some concern for employees.

Hypothesis 4 proposes a positive relationship between teamwork climate and SOB, which was supported by the data. The COVID-19 pandemic has introduced psychological isolation that impaired the SOB of employees. However, these employees continue to engage in virtual teamwork. As a result, SOB may even be slightly enhanced in the WFH technological setup. This result contradicts the finding of Handke et al. [47], who found that virtual teamwork does not facilitate information exchange. Teammates are part of the company. If a particular employee is accepted by other colleagues, he or she would feel some sort of belonging to the company.

Hypotheses 5 and 6 propose that teamwork climate and SOB are related to work engagement. Both hypotheses were supported and were consistent with the findings of previous studies [49]. WFH via teamwork climate and SOB negatively affects work engagement. Work engagement also depends on some other factors including self-leadership and job autonomy. Self-leadership means that one might control their behaviour and perform a task accordingly. He or she usually look at his or her performance to see whether it is up to the prescribed standard. If an employee can have certain freedom to complete his or her job, work engagement will be increased [57]. Thus, as a remedy, employers should consider other factors that might affect work engagement positively in order to balance the negative effects caused by WFH.

Sense of belonging is a mediator between psychological isolation and work engagement. The same construct is also a mediator between teamwork climate and work engagement.

### 5.1. Theoretical Implications

Previous studies have highlighted the advantages of WFH [58]. For instance, employees could spend more time with their families instead of traveling back and forth from their homes to their offices. Therefore, these employees may be able to achieve better work–life balance. Employers also grant their employees additional flexibility in their work arrangements. WFH is not a new policy in United States and some European countries [58]. Although only few works have highlighted the disadvantages of WFH, this study provides empirical evidence suggesting that WFH negatively affects the work engagement of employees. Hong Kong is a good place for us to carry out this nature experiment. This is because almost all the companies in Hong Kong did not have the WFH policy before. We propose that the decrease in awareness and cognitive social presence pertaining to WFH situations, only minimally affects jobs that are task orientated in nature, as such jobs do not require a high level of social interaction. However, affective social presence may not be so easily established through virtual ways.

This study contributes to the existing literature by providing empirical evidence that supports the S-O-A theory. We explore the influence of some well-known variables on work engagement to expand our knowledge in this area. We also propose a mechanism that explains how physical isolation affects work engagement through psychological isolation, teamwork climate and SOB.

The role of communication is important in the work from home context [59]. The study starts off with physical isolation which is a tangible component, leading to psychological isolation, teamwork climate and sense of belonging which are intangible components. Finally, the destination is work engagement. Work engagement is a critical variable and affects work performance eventually.

### 5.2. Managerial Implications

Under such a challenging social environment, corporations should improve teamwork climate efficiency whilst promoting SOB and work engagement of employees, which, in turn, may promote organisational sustainability. Managers could demonstrate various technologies to their employees by organising seminars and demonstrations in physical and virtual settings. Through active staff engagements in virtual environments, employees could perceive the usefulness of online communication systems in promoting a teamwork climate. For instance, the breakout room function in Zoom can be used to hold small group meetings and discussions.

Given the lack of affective social presence in the WFH setup, virtual informal gatherings, such as virtual breakfasts, lunch or tea breaks where work-related matters are not discussed, could be arranged. New employees could be teamed up with individual mentors for informal support. Face-to face-meetings are encouraged with reference to the social distance guidance. Employees may require some emotional support from their mentors and colleagues.

### 5.3. Limitations and Future Research Implications

The proposed model includes several constructs with sufficient explanatory power for determining work engagement. Future research may consider other factors to predict work engagement under unpredictable situations. For instance, a considerable number of employees have been asked to work from home and meet their colleagues by using virtual tools amidst the COVID-19 outbreak. Their experiences and intention to continue using these systems may affect future work policies and arrangements. Further research should explore the communication systems used for work and evaluate their power in explaining the intention of employees to continue using these systems.

Our quantitative research investigates the relationships amongst the physical isolation, psychological isolation, teamwork climate, SOB and work engagement of employees. However, the reasons that underlie psychological isolation and SOB are yet to be further investigated. Qualitative research may be conducted in the future to explore those attributes that can lead to SOB and teamwork climate.

We have not investigated the background and web technology competence of employees. Future research may explore the potential influence of gender, character, mindset, self-motivation and web technology experiences on the psychological isolation of employees over an extended period of time. Cultural characteristics of the respondents would have an impact on teamwork style. A cross-country study is encouraged to contrast the difference between the East and West.

The association between physical isolation and psychological isolation might need further research. The association could be different between a newcomer and an experienced executive. A newcomer needs more personal coaching and caring. Experienced executive is an independent staff. We expect that the association would be very weak for experienced executive.

## 6. Conclusions

This research applies the S-O-A theory to assess the work engagement of employees under an unforeseeable stimulus, such as the WFH policy in Hong Kong. Whilst certain technologies, such as video conferencing software, can solve most communication problems encountered in the office, having remote access and control of office computers can also help employees fulfil most of their office tasks. Company operations are not affected by working from home. However, corporations should organise virtual team building workshops given their significant role in facilitating a teamwork climate. The findings of this work verify the disadvantages of the WFH arrangement from the perspective of the S-O-A theory.

Given the influence of an unforeseeable stimulus, such as the COVID-19 pandemic, on businesses and industries, the adoption of WFH measures is considered inevitable. This study assesses the work engagement of employees in the WFH context. Our study examines how WFH affects 206 employees amidst the COVID-19 pandemic. The proposed model explains almost half of the variances for work engagement. Therefore, this model can provide a preliminary understanding of the effect of WFH on work engagement. In terms of practical implications, this study highlights the continuing significance of organising events for building team spirit and engaging employees. Through blended means of virtual means and secure human-to-human interactions within an organisational setting during the arrangement of WFH under a pandemic situation is an obvious consideration. Organisational leaders have the inevitable role of encouraging and facilitating staff engagement activities to enhance organisational sustainability in challenging operating environments [10].

## Figures and Tables

**Table 1:** Demographic data of respondents.

Category		Frequency	Percentage %
Gender	Male	110	53.4
	Female	96	46.6
Age	18-30	80	38.8
	31-40	40	19.4
	41-50	64	31.1
	51-60	18	8.7
	61 or above	4	1.9
Company size	Less than 5 people	16	7.8
	5–20 persons	28	13.6
	21–50 persons	40	19.4
	51–100 persons	40	19.4
	101 or above	82	39.8
Industry	Tourism	0	0
	Financial services	32	15.5
	Civil servant	14	6.8
	Retailing and customer services	34	16.5
	Trading & Logistics	26	12.6
	Professionals	84	40.8
	Cultural and creative	8	3.9
	Others	8	3.9
Tenure	Less than 6 months	20	8.7
	6 months to less than 2 years	60	32.0
	2–5 years	60	22.3
	5–10 years	60	13.6
	10 years or above	90	23.3
Job level	Entry level	96	38.8
	Supervisory level	80	27.2
	Middle management level	60	25.2
	Senior management level	30	6.8
	Director level	24	1.9

**Table 2:** Measurement Model Assessment.

Construct	Item	Loading	Cronbach’s Alpha	Composite Reliability	AVE
Psychological isolation	PSI1	0.695	0.897	0.919	0.619
	PSI2	0.795			
	PSI3	0.787			
	PSI4	0.719			
	PSI5	0.856			
	PSI6	0.850			
	PSI7	0.828			
Teamwork	TW1	0.715	0.814	0.878	0.645
	TW4	0.886			
	TW5	0.862			
	TW6	0.737			
Sense of belonging	SB1	0.792	0.797	0.866	0.618
	SB3	0.738			
	SB4	0.824			
	SB6	0.787			
Work Engagement	WE2	0.763	0.905	0.924	0.605
	WE3	0.863			
	WE4	0.772			
	WE5	0.753			
	WE6	0.747			
	WE7	0.887			
	WE8	0.788			
	WE9	0.676			

**Table 3:** Assessing Discriminant Validity (HTMT).

Construct	Mean	Standard Deviation	Physical Isolation	Psychological Isolation	Sense of Belonging	Teamwork	Work Engagement
Physical Isolation	2.630	1.963					
Psychological Isolation	4.366	1.398	0.261				
Sense of Belonging	3.740	1.336	0.145	0.592			
Teamwork Climate	4.374	1.149	0.139	0.214	0.645		
Work Engagement	4.100	1.152	0.126	0.192	0.577	0.722	

**Table 4:** Results of Hypotheses Testing.

Hypothesis	Item	(*β*) Path Coefficient	*t*-Value	*p*-Value	Result
H1	Physical isolation >> Psychological isolation	0.240	1.860	0.063 +	Supported
H2	Physical isolation >> Teamwork	−0.109	0.967	0.334	Unsupported
H3	Psychological isolation >> Sense of belonging	−0.394	3.919	0.000 ***	Supported
H4	Teamwork >> Sense of belonging	0.498	6.220	0.000 ***	Supported
H5	Teamwork >> Work Engagement	0.550	6.515	0.000 ***	Supported
H6	Sense of belonging >> Work Engagement	0.233	2.143	0.032 *	Supported

(Bootstrap samples = 5000, *n* = 206 cases). + *p* < 0.1; * *p* < 0.05; *** *p* < 0.001.

## Data Availability

Data are available upon request.

## Appendix A

Physical isolation (adapted from Bartel et al., 2012)

Physical isolation could be measured by a single item that is how many days per week on average you are engaged in home office mode from August this year.

0 day

1 day

2 days

3 days

4 days

5 days

6 days

7 days

Psychological isolation (adapted from Golden et al., 2008)

1. I feel left out on activities and meetings that could enhance my career.
2. I miss out on opportunities to be mentored.
3. I feel out of the loop.
4. I miss face to face contact with co-workers.
5. I feel isolated.
6. I miss the emotional support of co-workers.
7. I miss informal interaction with others.

Work Engagement (adapted from Schaufeli et al., 2006)

1. At my work, I feel bursting with energy.
2. At my job, I feel strong and vigorous.
3. I am enthusiastic about my job.
4. My job inspires me.
5. When I get up in the morning, I feel like going to work.
6. I feel happy when I am working intensely.
7. I am proud of the work that I do.
8. I am immersed in my work.
9. I get carried away when I am working.

Sense of Belonging (adapted from William, 2003)

1. I feel like an outsider (reverse scale).
2. I make friends easily.
3. I feel like I belong.
4. I feel awkward and out of place (reverse scale).
5. Other colleagues seem to like me.
6. I feel lonely (reverse scale).

Teamwork climate (adapted from Sexton et al., 2017)

1. My input is well received in workplace.
2. It is difficult to speak up in my workplace (reverse scale).
3. Disagreements in my workplace are appropriately resolved.
4. I have the support if I need from my colleagues.
5. It is easy to ask questions if I do not understand how to do it.
6. My colleagues and I work together as a well-coordinated team.
